# Thermal comfort, health, and performance effects among outdoor workers in northern Sweden

**DOI:** 10.1093/annweh/wxag025

**Published:** 2026-04-17

**Authors:** Rebecca Tapper, Edit Zimmerman, Hans Pettersson, Tiina Maria Ikäheimo, Jens Wahlström, Albin Stjernbrandt

**Affiliations:** Department of Epidemiology and Global Health, Umeå University, Umeå 901 87, Sweden; Department of Epidemiology and Global Health, Umeå University, Umeå 901 87, Sweden; Department of Epidemiology and Global Health, Umeå University, Umeå 901 87, Sweden; Department of Community Medicine, UiT, The Arctic University of Norway, Tromsø, Norway; Research Unit of Population Health, University of Oulu, Oulu, Finland; Department of Epidemiology and Global Health, Umeå University, Umeå 901 87, Sweden; Department of Epidemiology and Global Health, Umeå University, Umeå 901 87, Sweden

**Keywords:** occupational, cold climate, temperature, child day care centers, parks and recreational, electric wiring, risk management, Raynaud's phenomenon, respiratory symptoms, musculoskeletal symptoms

## Abstract

**Objectives:**

To investigate subjective differences in thermal comfort, health, and performance outcomes between different outdoor professions and the sexes. The current study also aimed to explore whether thermal comfort affected health and performance outcomes.

**Method:**

A questionnaire was used to collect data from three different professions: preschool (*n* = 65), park and maintenance (*n* = 36), and power grid workers (*n* = 31). The questionnaire contained items related to background variables (sex, age, body mass index, tobacco use, pre-existing diseases, and clothes provided by the employer), thermal comfort, self-assessed health, and performance outcomes.

**Results:**

The findings show that women, to a greater extent than men, experience cold-related discomfort while working in cold environments. Cold sensitivity was most common among park and maintenance workers (57%) and power grid workers (47%). Raynaud’s phenomenon and abnormal cold sensitivity were most common among park and maintenance workers, with a prevalence of 31% and 56%, respectively. Statistically significant differences were also found in all reported performance outcomes (concentration, endurance, mobility, strength, and speed) between the different professions. Associations between cold-related discomfort and an increased reporting of health symptoms and a decrease in performance outcomes were found.

**Conclusion:**

There were differences in perceived thermal comfort, health, and performance outcomes between different outdoor professions and sexes. The associations between cold-related discomfort, cold-related symptoms, and decreased performance indicate a need to implement cold risk management to ensure a safe work environment.

What's Important About This Paper? By describing thermal comfort, health and performance outcomes from ambient cold exposure, this study shows differences regarding sex and professions with high reporting of cold-related symptoms, cold sensitivity and decreased performance. The findings highlight the need for awareness and implementation of cold risk management for the sustainability and safekeeping of outdoor workers in cold environments.

## Introduction

Cold exposure can be defined as an uncomfortable sensation of cool or cold, which can occur at ≤10 °C (ISO 15743:2008). Most of the population in the Nordic countries is exposed to temperatures below this temperature daily during the winter (Hassi et al. 2002), and more than one-third of the Swedish workforce is regularly exposed to cold climatic conditions during working hours (Swedish Work Environment Authority 2025). A person can be exposed to cold via cold air, immersion in cold water, or by touching cold surfaces (Holmér 2001; Mäkinen 2007). Cold exposure may evoke pain, loss of sensation, rigidity, and decreased grip strength (Carlsson 2017), but also reduces work ability, decreases physical and mental performance (Holmér 1993; Raatikka et al. 2007), and increases the risk of accidents at the workplace (Hassi et al. 2002; Mäkinen and Hassi 2009). Low temperatures can also increase overall morbidity (Wang et al. 2021) and mortality (The Eurowinter Group 1997; Gasparrini et al. 2015) through cardiovascular, respiratory, and infectious diseases (Näyhä 2005). Body cooling primarily depends on the environment, the level of physical activity, and the type of clothing used (Mäkinen and Hassi 2009; Heil et al. 2016).

Thermal comfort is related to the person's whole-body thermal balance, which is influenced by factors such as physical activity, clothing, and environmental parameters (air temperature, mean radiant temperature, air velocity, and humidity) (ISO 7730:2025). When exposed to local cooling or heating, such as touching cold or hot surfaces or objects, thermal discomfort might occur, especially if proper clothing insulation is lacking (ISO 11079:2007; ISO 13732-3:2005; ISO 15265:2004; ISO 28802:2012). Although clothing properties, insulation required, and duration-limited exposure models have been developed and used (ISO 11079:2007; ISO 9920:2007), assessing cold environments remains challenging (d’Ambrosio Alfano et al. 2013, 2025). Moreover, a new ISO standard is currently being developed that will encompass adaptive methods for achieving thermal comfort (ISO/TR 23672). Individual responses to exposure can vary and are influenced by physiological factors such as age, sex, fitness, body size, health, and body mass index (BMI), as well as psychological and contextual factors such as cultural or situational norms, mood, and personal expectations (Carlsson 2017; Stjernbrandt et al. 2018; Naheed and Shooshtarian 2021; Özbey and Turhan 2024).

Thermal sensation relates to the subjective experience of thermal comfort and is described as the conscious subjective expression of one's thermal perception of the environment, which is captured by subjective voting from cold to hot (ISO 28802:2012; Yang et al. 2024). Psychological and subjective experiences of thermal comfort have been shown to reflect the thermal sensations more accurately, and psychological and behavioral adaptations seem to be an important factor (Feng et al. 2023; Mao et al. 2023).

Previous research has shown that women experience discomfort from cold at higher temperatures and more during the winter season compared with men (Karjalainen 2007; Vellei et al. 2025). Various occupational groups, such as food industry, construction, or traffic controls has been studied regarding cold exposure and associated health aspects and thermal sensation (Aasmoe et al. 2008; Inaba et al. 2009; Burström et al. 2010; Oliveira et al. 2014a, 2014b), as well as exposure to cold indoor temperatures (Janssen et al. 2023). Differences in physical activity and different work tasks have also been studied as an influence on the thermal responses and work capacity (Sormunen et al. 2009; Foster et al. 2021; Zhang et al. 2025). However, knowledge is lacking regarding how outdoor occupational cold exposure affects common health conditions, thermal sensations and performance outcomes in three common outdoor occupational settings: preschool, park and maintenance, and power grid workers. Our previous study among these three occupations demonstrated that the daily exposure to outdoor cold, for these professions, was on average 2 h or more, and that a third of the workers indicated that they often or always felt cold at work (Tapper et al. 2025).

The current study, therefore, aimed to describe differences in subjective thermal comfort, as well as self-reported health and performance outcomes between different professions involving outdoor exposure (preschool, park and maintenance, and power grid workers), as well as between sexes. It also aimed to explore whether subjective thermal comfort is associated with poorer self-reported health and performance outcomes.

## Methods

### Study design and participants

This cross-sectional study included participants via convenience sampling of workers who regularly worked outdoors. The data was attained during one winter season, between November 2023 and April 2024. It was collected from three different professions: preschool, park and maintenance, and power grid workers in northern Sweden. Two of the chosen professions were either female- (preschool) or male-dominated (power grid) and one had a more equal distribution between sexes (park and maintenance). In northern Sweden, these three professions conduct outside work tasks daily, all year round, in various kinds of weather. For example, preschool personnel organize and supervise outdoor playtime, power grid workers maintain the structural integrity of overhead power lines and switchgear, and park and maintenance workers clear snow and arrange outdoor lights. Different workplaces were enrolled via human resources partners within the organizations or by first-line managers. Information was given via phone, e-mail, and an oral presentation about the project during staff meetings by one of the researchers (R.T.). If the workplaces were too far away, information was provided in a pamphlet that the managers distributed. To obtain a large enough sample, recruitment was conducted at three to six different workplaces in each occupational sector, and the number of employees in each workplace ranged from 7 to 30 workers. A day for data collection was decided on between the managers and the researcher.

### Data collection and variables

The data were collected and managed using REDCap electronic data capture tools hosted at Umeå University (Harris et al. 2009, 2019). Participants were asked to fill out at the end of their workday. Reminder e-mails and text messages were sent out after a week to those who had not responded. The survey consisted of 50 questions regarding occupation, self-assessed health, subjective experiences of working in a cold environment, perceived exertion and cold risk management (Appendix S3). The questions were based on the ISO standard for cold assessment (15743:2008) and the biennial work environment survey by the Swedish Work Environment Authority (2022).

### Individual variables

The questionnaire contained background questions such as age, sex, height, and weight, which were used to calculate BMI and categorized into three groups based on cut-off points (Arif and Weir 2019). Questions regarding the use of tobacco, local cold injuries (frostbites) and physician-diagnosed hypertension, coronary heart disease, asthma, chronic obstructive pulmonary disease (COPD), rheumatic disease, diabetes, and osteoarthritis were asked. The participants were also asked what type of protective clothing the employer provided against cold exposure.

### Thermal comfort

Thermal comfort was assessed by asking, “*How do you usually experience the thermal comfort when working outdoors in the winter?”* regarding the whole body, face, hands, and feet. For each body part, the participant could answer from cold to warm on a five-level Likert-type scale: “*very warm,” “slightly warm,” “neutral,” “slightly cold,”* and “*very cold.”* For analyses, “*very warm”* and “*slightly warm”* were grouped as “*warm,”* while “*very cold”* and “*slightly cold”* were grouped as “*cold.”*

### Health outcomes

Health effects from cold exposure were inquired from the participants, checking a specific box for each symptom that they experienced. The following question was asked in Swedish and directly translated to: “*Do you experience any of the following problems when you are in the cold?.”* The respiratory symptoms were: “*shortness of breath,” “cough,” “wheezing,” “increased mucus production in the airways,” “nasal congestion,”* and “*runny nose.”* The musculoskeletal symptoms were: “*pain in neck/shoulders,” “pain in arms,” “pain in thoracic/lumbar spine,”* “*pain in hips,”* and “*pain in knees.”*

For the analyses, respiratory and musculoskeletal symptoms were combined into single binary variables. Each was considered positive if at least one of the symptoms mentioned above was acknowledged.

The occurrence of Raynaud’s phenomenon was assessed by showing the participants a color image as an example (Maricq and Weinrich 1988) and asking: “*Does one or more of your fingers turn white (as shown on the picture) when exposed to moisture or cold?”* to which the participant could answer yes or no. Cold sensitivity was asked for using the following question: “*Cold sensitivity in the hands is defined as a collection of acquired symptoms (pain, sensory alterations, stiffness, or color changes) that result in an abnormal aversion to cold. Do you experience any of this?”* (Campbell and Kay 1998). The answers were: “*not at all,” “hardly at all,” “a little,” “quite a lot,”* and “*very much.”* For analyses, a binary variable was used where “*a little,”* “*quite a lot,”* or “*very much”* was considered a positive response.

### Performance outcomes

The participants were asked if working in the cold affected any of the following factors negatively: “*concentration,” “endurance,” “mobility,” “strength,”* and *“speed.”* For each factor, the participants could answer a five-level Likert scale from “*not at all” to “very much.”* For the analyses, a binary variable was created where *“a little,” “quite a lot,”* or “*very much”* were considered a positive response.

### Statistical analysis

The chi-square test was used to evaluate potential differences in health outcomes, performance outcomes, and thermal comfort between the professions (pairwise) and between sexes. Additional analyses were conducted within the park and maintenance group due to the equal distribution between the sexes. When the sample sizes were too small (*n* < 5), Fisher's exact test was used instead. Binary logistic regression was performed to explore associations between thermal comfort and health and performance outcomes. Statistical analyses were performed using IBM SPSS Statistics for Windows (Version 29.0, IBM Corporation, Armonk, NY, USA).

### Ethics

The study was approved by the Swedish Ethical Review Authority *(Dnr2023-04373-01)* and performed in accordance with the ethical principles outlined in the Declaration of Helsinki. All subjects provided signed consent to participate.

## Results

### Descriptive data

The study population consisted of 78 (59%) women and 54 (41%) men. Workers within preschools consisted of 92% women. Within park and maintenance, 47% of the workers were women, and within power grid, 3% were women. The mean age was 41(sd = 13) years, mean BMI 26 (sd = 4), and 29% of the study population were currently using tobacco. The prevalence of frostbite, at any location, for the entire study population was 51%. The most common location of frostbite was the face (21%), followed by the hands (16%). Previously physician-diagnosed conditions included hypertension (13%) and asthma (14%) as the most common, for the entire study population. Furthermore, results show that it was most common among power grid workers to receive full protective clothing (ie jacket, trousers, gloves, and boots) from their employer, and the least common among preschool workers (Table 1). Only 15% of the preschool workers were provided with shoes by the employer, compared with 97% of the park and maintenance, and power grid workers.

**Table 1:** Characteristics of the study population.

Variable	Category	Preschool	Park and maintenance	Power grid	Total
		*n* (%)	*n* (%)	*n* (%)	*n* (%)
**Sex**	Female	60 (92)	17 (47)	1 (3)	78 (59)
	Male	5 (8)	19 (53)	30 (97)	54 (41)
**Age (years)**	<30	18 (29)	2 (6)	11 (36)	31 (24)
	31 to 40	20 (32)	8 (22)	6 (19)	34 (26)
	41 to 50	12 (19)	12 (33)	8 (26)	32 (25)
	>51	13 (21)	14 (39)	6 (19)	33 (25)
**BMI (kg/m^2^)**	<25	32 (58)	15 (42)	8 (26)	55 (45)
	25 to 30	17 (31)	18 (50)	10 (32)	45 (37)
	>30	6 (11)	3 (8)	13 (42)	22 (18)
**Tobacco use**	Current	16 (25)	10 (29)	12 (39)	38 (29)
	Previous	4 (6)	1 (3)	3 (10)	8 (6)
	Never	45 (70)	24 (69)	16 (52)	85 (65)
Diseases*	Hypertension	7 (11)	5 (14)	5 (16)	17 (13)
	Coronary heart disease	2 (3)	0 (0)	1 (3)	3 (2)
	Asthma	11 (18)	4 (11)	3 (10)	18 (14)
	Rheumatic disease	2 (3)	0 (0)	0 (0)	2 (2)
	Osteoarthritis	4 (7)	4 (11)	2 (7)	10 (8)
	Frostbite face	7 (11)	7 (21)	12 (41)	26 (21)
	Frostbite hands	5 (8)	9 (27)	6 (21)	20 (16)
	Frostbite feet	9 (15)	6 (18)	0 (0)	15 (12)
	Frostbite other	2 (3)	2 (6)	2 (7)	6 (5)
**Clothes provided by the employer**	Gloves	51 (79)	35 (97)	31 (100)	117 (89)
	Shoes	10 (15)	35 (97)	30 (97)	75 (57)
	Jacket	55 (85)	34 (94)	31 (100)	120 (91)
	Thermal pants	53 (82)	32 (89)	26 (84)	111 (84)
	Coverall	7 (11)	6 (17)	16 (52)	29 (22)
	Hat	32 (49)	31 (86)	31 (100)	94 (71)
	Other	7 (11)	4 (11)	8 (26)	19 (14)
	None	4 (6)	1 (3)	0 (0)	5 (4)

^*^Chronic obstructive pulmonary disease and diabetes were included, but not reported by any subjects.

### Differences in thermal comfort

There was a statistically significant difference in whole body thermal comfort, thermal comfort in the face, and thermal comfort in the feet between the different professions (Table 2). A larger proportion of preschool workers (41%) reported that they felt cold in their whole body compared with power grid workers (10%). It was most common for park and maintenance workers (81%) to report that they felt cold in the face compared with preschool workers (71%) and power grid workers (39%). For thermal comfort in the feet, the same trend was observed, where 45% of the power grid workers felt that it was cold compared with 73% of the preschool workers and 80% of the park and maintenance workers (Table 2).

**Table 2:** Differences in thermal comfort between the professions and sexes.

Thermal comfort in:	Category	Preschool	Park and maintenance	Power grid		Female	Male	
		*n* (%)	*n* (%)	*n* (%)	*P* value	*n* (%)	*n* (%)	*P* value
**Whole body**	Neutral	26 (41)	11 (31)	12 (39)	0.003^a^	25 (33)	24 (44)	<0.001
	Warm	11 (18)	13 (37)	16 (52)		15 (20)	25 (46)	
	Cold	26 (41)	11 (31)	3 (10)		35 (47)	5 (10)	
**Face**	Neutral	14 (22)	5 (14)	14 (45)	0.005^F,a,b^	14 (18)	19 (35)	0.014^F^
	Warm	4 (6)	2 (6)	5 (16)		4 (5)	7 (13)	
	Cold	45 (71)	29 (81)	12 (39)		58 (76)	28 (52)	
**Hands**	Neutral	12 (19)	6 (17)	10 (32)	0.158^F^	12 (16)	16 (30)	0.125^F^
	Warm	6 (10)	0 (0)	2 (7)		4 (5)	4 (7)	
	Cold	45 (71)	29 (83)	19 (61)		59 (79)	34 (63)	
**Feet**	Neutral	11 (17)	6 (17)	9 (29)	0.013^F,a,b^	11 (15)	15 (28)	0.001
	Warm	6 (9)	1 (3)	8 (26)		4 (5)	11 (20)	
	Cold	47 (73)	28 (80)	14 (45)		61 (80)	28 (52)	

^F^Fisher’s exact test.

^a^Significant difference between preschool and power grid workers.

^b^Significant difference between park and maintenance workers and power grid workers.

Between sexes, statistically significant differences were found in whole body thermal comfort, thermal comfort in the face, and thermal comfort in the feet, where women, to a greater extent, reported cold discomfort at work compared with men (Table 2). When only testing sex differences among park and maintenance workers, a statistically significant difference was found for whole body thermal comfort, where women (56%) more often felt that it was cold when working compared with 11% of the men.

### Differences in health outcomes

The occurrence of Raynaud's phenomenon differed significantly between the three different professions, and it was most common among park and maintenance workers (31%) compared with preschool workers (13%) and power grid workers (10%) (Table 3). There was also a statistically significant difference between the three professions concerning cold sensitivity, where it was least common among preschool workers (25%) compared with park and maintenance workers (57%) and power grid workers (47%).

**Table 3:** Differences in perceived negatively affected health outcomes between the professions and the sexes.

Health outcome	Preschool	Park and maintenance	Power grid		Female	Male	
	*n* (%)	*n* (%)	*n* (%)	*P* value	*n* (%)	*n* (%)	*P* value
**Respiratory symptoms**	42 (65)	23 (64)	18 (58)	0.816	54 (54)	29 (69)	0.069
**Musculoskeletal symptoms**	15 (23)	10 (28)	4 (13)	0.326	21 (27)	8 (15)	0.099
**Raynaud's phenomenon**	8 (13)	11 (31)	3 (10)	0.009^a,b^	16 (21)	6 (11)	0.136
**Cold sensitivity**	15 (25)	20 (56)	14 (47)	0.035^a,c^	26 (36)	23 (43)	0.410

^a^Significant difference between preschool and park and maintenance workers.

^b^Significant difference between park and maintenance workers and power grid workers.

^c^Significant difference between preschool and power grid workers.

No statistically significant difference between the sexes was found for respiratory or musculoskeletal symptoms, Raynaud's phenomenon or cold sensitivity (Table 3).

### Differences in performance outcomes

Performance (concentration, endurance, mobility, strength, and speed) varied significantly between preschool workers, who reported being less affected by the cold, compared with park and maintenance workers, as well as differences in concentration and strength between preschool and power grid workers (Table 4).

**Table 4:** Differences in perceived negatively affected performance variables between the professions and sexes.

Performance outcome	Preschool	Park and maintenance	Power grid		Female	Male	
	*n* (%)	*n* (%)	*n* (%)	*P* value	*n* (%)	*n* (%)	*P* value
**Concentration**	12 (19)	15 (44)	12 (39)	0.017^a,b^	21 (28)	18 (34)	0.441
**Endurance**	26 (41)	27 (75)	18 (58)	0.005^a^	41 (54)	30 (56)	0.856
**Mobility**	36 (56)	31 (86)	23 (74)	0.006^a^	50 (65)	40 (74)	0.267
**Strength**	21 (34)	19 (54)	18 (58)	0.040^a,b^	29 (39)	29 (54)	0.103
**Speed**	34 (55)	29 (83)	23 (74)	0.012^a^	47 (63)	39 (74)	0.195

^a^Significant difference between preschool and park and maintenance.

^b^Significant difference between preschool and power grid workers.

Regarding performance effects associated with cold, no differences between sexes were observed.

### Associations between thermal comfort and health and performance outcomes

Increased reporting of musculoskeletal symptoms was associated with cold-related discomfort within the whole body (OR 3.4; 95% CI 1.3 to 9.2), face (OR 6.7; 95% CI 1.5 to 30.2), and feet (OR 5.0; 95% CI 1.1 to 22.5). There was an increased reporting of cold sensitivity related to cold discomfort in the whole body (OR 3.6; 95% CI 1.4 to 9.1) and hands (OR 8.3; 95% CI 2.3 to 29.7) (Appendix S1). Cold-related discomfort in the whole body was associated with increased reporting of decreased concentration (OR 2.8; 95% CI 1.1 to 7.1) and mobility (OR 3.2; 95% CI 1.2 to 8.2) (Appendix S2).

## Discussion

### Main findings

This study showed that women reported cold-related discomfort to a greater extent than men while working in the cold. Power grid workers reported the lowest extent of cold-related discomfort compared with the other professions. It was also most common for power grid workers to receive full protective clothing from their employer. Cold sensitivity and Raynaud's phenomenon were most common among park and maintenance workers. Reduced performance when working in the cold was least reported by preschool workers. Associations between cold-related discomfort and increased reporting of musculoskeletal symptoms, cold sensitivity, and performance outcomes in terms of decreased concentration and mobility were found.

### Thermal comfort

This study showed that there were differences in thermal comfort between the professions as well as between sexes concerning all body parts considered except for the hands. Multiple reasons could influence these findings. Firstly, the nature of work tasks (activity rate, ambient temperature, and cold exposure duration) could be potential explanatory factors (Sormunen et al. 2009; Oliveira et al. 2014b). In accordance with our previous study (Tapper et al. 2025), which showed that 62% of the preschool workers’ outside time was spent standing still, compared with 39% for park and maintenance and 53% power grid workers. This could be one explanation for why a larger proportion of pre-school workers (most females) reported whole-body thermal discomfort compared with power grid workers (most males).

When performing additional analyses for park and maintenance workers, who had the most equal distribution between sexes, women still reported more cold-related discomfort. Several previous studies have explored thermal comfort differences between sexes (Karjalainen 2007; Lan et al. 2008; Liu et al. 2021), and a trend has been shown where women tend to experience more thermal discomfort at low temperatures compared with men (Karjalainen 2012; Liu et al. 2021). Cold discomfort and unpleasant sensations during the winter season have been found to be significantly more common among women than men (Karjalainen 2007). Women also seem to prefer a slightly warmer environment than men (Lan et al. 2008). Overall, this supports the notion that biological sex affects thermal comfort and should be considered in cold risk assessments.

Cold protective clothing and related equipment are also important determinants of thermal comfort. In our study, it was shown that preschool workers, and hence most of the females, had the least access to personal protective equipment (PPE) provided by the employer, which could be a result of the workplace culture and economic strains. Lack of access to PPE may be a contributing factor to the fact that females more often experience cold-related discomfort, as one of the most effective strategies is the ability to adjust clothing in cold environments (Oliveira et al. 2014a; Parsons 2021; Liu et al 2022). Additionally, there are reasons to suspect that other gender biases are influencing these findings, warranting further investigation. In particular, whether cold protective clothing might be better designed for male anatomy and comfort, or whether gendered disparities exist in offering adequate protective clothing across male and female-dominated workplaces.

### Health outcomes

Our study found that females reported a higher occurrence of Raynaud's phenomenon than males, and the condition was most prevalent among park and maintenance workers. Several previous studies have confirmed an association between cold exposure and Raynaud's phenomenon (Stjernbrandt et al. 2017, 2019, 2022). Others have also shown that it is more common among females (Roquelaure et al. 2012; Garner et al. 2015). Population studies conducted in both Sweden and Finland confirm this difference by sex (Raatikka et al. 2007; Stjernbrandt et al. 2022). Risk factors for Raynaud's phenomenon also include manual occupations (Garner et al. 2015) and exposure to hand-arm vibration (Burström et al. 2010; Nilsson et al. 2017). In our previous study, the duration of outdoor work in cold environments of these same professions was measured. Both park and maintenance, and power grid workers spend about half their day outside working (Tapper et al. 2025). However, the higher proportion of females working in park and maintenance, in combination with the risk factors previously mentioned, could explain why the prevalence of Raynaud's phenomenon was highest among park and maintenance workers and not power grid workers who had similar exposure duration to ambient cooling.

In a previous study on the Swedish working-age population, cold sensitivity was reported by about 14% of the women and 10% of the men (Stjernbrandt et al. 2017). In the current study, no significant difference was found between sexes regarding cold sensitivity, but the occurrence differed between the studied occupations.

Park and maintenance workers, as well as power grid workers, are generally outdoors at least half of their workday, which entails exposure to outdoor environments (Tapper et al. 2025). Previous studies have shown that intense cold exposure is associated with cold sensitivity (Stjernbrandt et al. 2017, 2018). Stjernbrandt et al. (2018) observed that previous frostbite in the hands increased the risk of cold sensitivity. Especially susceptible are people with traumatic hand injuries and hand-arm vibration injuries, where the occurrence of cold sensitivity is higher compared with healthy subjects (Carlsson et al. 2010). Power grid and park and maintenance workers, compared with preschool workers, are more likely to be exposed to hand-arm vibration via power tools (AFA försäkring 2023). These factors could explain why cold sensitivity was significantly more common among power grid, and park and maintenance workers.

We also found an association between cold-related discomfort in the whole body, face, and feet, and having musculoskeletal symptoms. This finding is supported by previous studies (Aasmoe et al. 2008; Farbu et al. 2022; Lewis et al. 2023), although not solely within outdoor settings. Previous studies have shown that there is an association between feeling cold at work and having musculoskeletal pain (Farbu et al. 2019). Also, seafood and food production studies have shown that the prevalence of musculoskeletal symptoms was high among cold-exposed workers, especially among female workers (Aasmoe et al. 2008). Cold discomfort and unpleasant sensations were significantly more common among female workers (Sormunen et al. 2009), supporting the notion that cold work is an important risk factor for musculoskeletal symptoms (Aasmoe et al. 2008) and that there is an association between musculoskeletal symptoms and poor work ability in cool working environments (Sormunen et al. 2009).

### Performance

Preschool workers reported that performance outcomes were less affected by cold exposure than those of other professions, although a larger proportion of preschool workers reported cold-related discomfort. This could perhaps be explained by the nature of the work tasks, differences in protective clothing, cognitive load or duration of cold exposure (Falla et al. 2021). Our results also showed that reporting whole-body cold discomfort was associated with decreased concentration and mobility. Previous studies (Cheung et al. 2016; Falla et al. 2021) have shown that cold exposure has a negative effect on cognitive functions (Falla et al. 2021), decision making, and task performance (Cheung et al. 2016). Whole-body cooling at work could also alter neuromuscular functions, decrease physical performance (Racinais and Oksa 2010), and, in this way, affect mobility. Mobility impairment can partly be explained by the large proportion of musculoskeletal symptoms (Lewis et al. 2023), or restricted movement due to having to use bulky protective clothing (Cheung et al. 2016).

### Methodological considerations

This study is part of a larger project (ArctiHealth) that includes multiple studies with a broader scope of capturing the effects of cold exposure and experiences across different occupational groups. For this reason, the data collection process was not limited to thermal comfort, health outcomes, or performance, yet all vital parts for understanding the complexity of cold exposure within different occupational sectors. There are several important limitations to consider. To begin with, our study only assessed subjective parameters of thermal comfort, health, and performance. Although worn clothing insulation is relevant for interpreting thermal perceptions, such was not recorded during the 2023 to 2024 measurements and cannot be reliably reconstructed retrospectively. Future studies should employ additional technical and quantitative measurements of environmental, microclimatic, and physiological parameters, such as individual metabolic rate and clothing thermal insulation, to provide a more robust foundation for the findings. Our study population was rather small and limited to workers in northern Sweden and three specialized occupational sectors. Moreover, data were only collected during one winter season (November 2023 to April 2024). These conditions limit the generalizability of the results. Increasing the sample size, expanding beyond Sweden, collecting data across different cold seasons, and including additional occupations would yield more generalizable and transferable results.

By constructing the questionnaire using questions from the ISO standard 15743:2008 and Swedish Work Environment Authority report 2022:2, including occupational groups that have not been previously investigated, the study adds knowledge on the association between cold exposure on reported health, thermal comfort, and performance between different professions and sexes. Some survey terms were slightly altered when translated between Swedish and English to improve clarity and context. For instance, thermal comfort was used in a manner closer to the term “temperature comfort.” It was thus considered a synonym for the feeling of being satisfied with the thermal environment (ISO 7730:2025).

### Practical implications

Firstly, our results show the importance of protecting workers from cold exposure as thermal comfort affects both health outcomes and performance. PPE must be adequate to ensure safety. Our findings show that pre-school workers and most of the female workers had limited access to complete protective clothing provided by their employer. Additionally, implementing organizational measures, such as offering additional rest periods in heated areas for recovery, could be a beneficial supplement. Secondly, due to the severity of health effects from cold exposure, we encourage employers to add cold exposure to their current work environment management plan, as performance and health are negatively affected by insufficient thermal comfort. Lastly, our results provide valuable information regarding these three specific professions in the northern regions of Sweden. This could be further developed and compared in future studies with cross-border collaborations within other Arctic areas. Finally, to further understand the long-term effects of cold exposure, longitudinal cohort studies are called for.

## Conclusion

Our study showed that there were differences in thermal comfort, as well as reported health and performance, between different professions and sexes related to exposure to cold in outdoor work. Our study also showed that there was an association between cold-related discomfort and higher reporting of cold-related symptoms, cold sensitivity, and decreased performance, indicating the need for cold risk management implementation to protect the workers.

## Supplementary Material

wxag025_Supplementary_Data

## Data Availability

De-identified questionnaire data can be shared upon reasonable request.
