# Supplementary material for: Thermal comfort, health, and performance effects among outdoor workers in northern Sweden
Source: Ann Work Expo Health. 2026 Apr 17;70(3):wxag025. doi: 10.1093/annweh/wxag025 (PMC13089684; doi:10.1093/annweh/wxag025)
Supplement: wxag025_Supplementary_Data [file wxag025_supplementary_data.pdf]

Thermal comfort, health, and performance effects among outdoor workers in northern Sweden

Rebecca Tapper[1], Edit Zimmerman[1], Hans Pettersson[1], Tiina Maria Ikäheimo[2,3], Jens Wahlström[1], Albin Stjernbrandt[1]

[1]Department of Epidemiology and Global Health, Umeå University, Sweden

[2] Department of Community Medicine, UiT, The Arctic University of Norway, Norway

[3] Research Unit of Population Health, University of Oulu, Finland

Corresponding author
Rebecca Tapper
Department of Epidemiology and Global Health
Umeå University
901 87, Umeå, Sweden
E-mail: rebecca.tapper@umu.se

ORCID:

Rebecca Tapper 0009-0009-8193-1352

Hans Pettersson 0000-0001-7077-2389

Jens Wahlström 0000-0002-2359-509X

Tiina Ikäheimo 0000-0002-2763-6004

Albin Stjernbrandt 0000-0001-6082-8465

Word count: 4053 (including in text references)

Appendix 1. Associations between thermal comfort and health outcomes

**Thermal comfort in:**	**Category**	**Respiratory symptoms**	**Musculoskeletal symptoms**	**Raynaud's phenomenon**	**Cold sensitivity**
		**OR (95% CI)**	**OR (95% CI)**	**OR (95% CI)**	**OR (95% CI)**
Whole body	Neutral (ref.)				
	Warm	0.9 (0.4-2.2)	0.7 (0.2-2.4)	1.8 (0.5-6.3)	1.8 (0.7-4.6)
	Cold	2.1 (0.8-5.2)	3.4 (1.3-9.2)*	3.0 (0.9-9.6)	3.6 (1.4-9.1)*
Face	Neutral (ref.)				
	Warm	0.3 (0.1-1.2)	1.6 (0.1-19)	0.6 (0.1-5.4)	0.8 (0.2-3.3)
	Cold	1.8 (0.8-4.1)	6.7 (1.5-30.2)*	1.3 (0.4-3.9)	0.9 (0.4-2.1)
Hands	Neutral (ref.)			= 0	
	Warm	0.7 (0.1-3.5)	0.9 (0.08-8.96)		2.8 (0.4-20.5)
	Cold	2.8 (1.2-6.7)*	2.1 (0.7-6.6)		8.3 (2.3-29.7)*
Feet	Neutral (ref.)				
	Warm	0.98 (0.3-3.5)	0.9 (0.07-10.3)	0.5 (0.05-5.6)	2.2 (0.6-8.8)
	Cold	1.8 (0.7-4.3)	5.0 (1.1-22.5)*	1.9 (0.5-7)	2.6 (0.95-7.2)

* Statistically significant results (p value < 0.05).

Appendix 2. Associations between thermal comfort and performance outcomes

**Thermal comfort in:**	**Category**	**Concentration**	**Endurance**	**Mobility**	**Strength**	**Speed**
		**OR (95% CI)**	**OR (95% CI)**	**OR (95% CI)**	**OR (95% CI)**	**OR (95% CI)**
Whole body	Neutral (Ref.)					
	Warm	1.6 (0.6-4.3)	0.7 (0.3-1.5)	2.4 (0.98-6.1)	1.4 (0.6-3.2)	1.5 (0.6-3.8)
	Cold	2.8 (1.1-7.1)*	2.2 (0.9-5.3)	3.2 (1.2-8.2)*	1.9 (0.8-4.4)	1.5 (0.6-3.7)
Face	Neutral (Ref.)					
	Warm	1.3 (0.3-5.5)	0.7 (0.2-2.8)	0.3 (0.1-1.3)	1.1 (0.3-4.3)	1.2 (0.3-5.6)
	Cold	0.9 (0.4-2.3)	2.0 (0.9-4.4)	0.9 (0.4-2.2)	1.1 (0.5-2.6)	0.9 (0.4-2.3)
Hands	Neutral (Ref.)					
	Warm	1.2 (0.2-7.7)	0.3 (0.1-1.9)	0.7 (0.1-3.1)	0.8 (0.2-3.6)	0.4 (0.1-2.2)
	Cold	1.7 (0.6-4.8)	1.5 (0.6-3.4)	1.8 (0.8-4.5)	0.6 (0.2-1.3)	1 (0.4-2.6)
Feet	Neutral (Ref.)					
	Warm	2.2 (0.6-8.8)	0.6 (0.2-2.1)	1.7 (0.4-6.9)	2.3 (0.6-8.8)	1.1 (0.3-4.3)
	Cold	1.4 (0.5-3.8)	1.2 (0.5-2.8)	1.6 (0.6-3.9)	0.9 (0.4-2.1)	1.2 (0.5-3.1)

* Statistically significant results (p value < 0.05).

Appendix 3. Study survey, translated from Swedish to English

ArctiHealth – a project on experiences of cold exposure, health, and the prevention of cold-related risks in working life

Date: ___________

Name: __________________

Date of birth (YYMMDD): ________________

Gender:

- Male
- Female
- This classification does not apply to me

How long have you lived in northern Sweden?
(Norrbotten, Västerbotten, Jämtland-Härjedalen or Västernorrland)

- Less than 1 year
- 1–5 years
- 6–10 years
- 11–20 years
- More than 20 years
- Do not know / prefer not to answer

Height (cm): __________________

Weight (kg): __________________

What is your highest level of education?

- Compulsory school (years 1–9)
- Gymnasium
- University
- Do not know / prefer not to answer

Do you use tobacco (cigarettes, snus, e-cigarettes, or similar) in any form?

- Yes, currently
- Yes, previously
- No
- Do not know / prefer not to answer

How often have you exercised in sportswear during the past three months, with the aim of improving your fitness or well-being?

- Never
- Occasionally, not regularly
- Once per week
- Two to three times per week
- More than three times per week
- Do not know / prefer not to answer

In general, how would you rate your health?

- Excellent
- Very good
- Good
- Fair
- Poor
- Do not know / prefer not to answer

Occupation (e.g. carpenter, childcare worker): __________________

Workplace (e.g. Charles' construction company): __________________

How many hours do you usually work per week?

- 0–10 hours per week
- 11–20 hours per week
- 21–30 hours per week
- 31–40 hours per week
- More than 40 hours per week
- Do not know / prefer not to answer

How many years have you worked in your current occupation?

- Less than 1 year
- 1–5 years
- 6–10 years
- 11–20 years

- More than 20 years
- Do not know / prefer not to answer

Assuming that your work ability at its best is rated as 10 points, how would you rate your current work ability?

- 0 (Unable to work)
- 1
- 2
- 3
- 4
- 5
- 6
- 7
- 8
- 9
- 10 (Best possible work ability)

How physically demanding has your work been during the past week?

- 6 (No exertion at all)
- 7 (Extremely light)
- 8
- 9 (Very light)
- 10
- 11 (Light)
- 12
- 13 (Somewhat demanding)
- 14
- 15 (Demanding)
- 16
- 17 (Very demanding)
- 18

- 19 (Extremely demanding)
- 20 (Maximally demanding)

The following questions concern the work environment at your workplace.

Do you talk at your workplace about how the working environment can be improved?

- Very much
- Quite a lot
- To some extent
- Hardly at all
- Not at all
- Do not know / prefer not to answer

Do you feel encouraged to contribute ideas and suggestions for improvements related to your work?

- Very much
- Quite a lot
- To some extent
- Hardly at all
- Not at all
- Do not know / prefer not to answer

The following questions concern your experience of working in cold conditions.

What proportion of your working time are you usually exposed to cold?

- Not at all
- One tenth of the time
- One quarter of the time
- Half of the time
- Three quarters of the time
- Almost all of the time
- Do not know / prefer not to answer

On average, how many hours per day are you usually exposed to cold OUTSIDE working hours?

- Less than 1 hour
- 1–2 hours
- 3–4 hours
- 5–6 hours
- More than 6 hours

(Winter season; outdoors below +10 °C, e.g., walking, snowmobiling, skiing, or artificial cold such as time spent in an ice rink)

Are you exposed to other conditions at work that make you feel cold?

Wind/draughts	
Moisture/precipitation	
Contact with cold objects	
Other factors (e.g. work situations where protective clothing cannot be used)	
No, none	
Do not know / prefer not to answer	

(Multiple responses possible)

Please specify which other conditions at your workplace make you feel cold:

____

During a typical working day, do you experience feeling cold?

- Never
- Very rarely
- Rarely
- Sometimes
- Often
- Always

If your work requires you to remain in cold environments, for approximately how long do you usually work continuously in the coldest environment?

- Less than 1 hour

- 1–3 hours
- 4–5 hours
- More than 5 hours
- I do not work in cold environments
- Do not know / prefer not to answer

This section focuses on the management of cold exposure.

Do you feel that you have knowledge about how to protect yourself against cold?

- Yes
- Yes, to some extent
- No, hardly
- No, not at all
- Do not know / prefer not to answer

Are there heated facilities at your workplace where you can take breaks and recover?

- Completely agree
- Partly agree
- Agree to a small extent
- Do not agree at all
- Do not know / prefer not to answer

If your work allows breaks in a warm environment, approximately how long is each break?

- Less than 15 minutes
- 15–30 minutes
- 31–45 minutes
- 46–60 minutes
- More than 60 minutes
- Do not know / prefer not to answer

What measures do you usually take when you feel cold at work?

Put on more clothing	

Increase physical activity	
Move to a warmer environment	
Change work tasks	
Stop working	
Do not take any measures	
Other	

(Multiple responses possible)

Please specify other measures:

____

What types of cold-protective work clothing are you provided with by your employer?

None	
Gloves/mittens	
Footwear	
Jacket/coat	
Over-trousers	
Overall/coverall	
Hat	
Other	
Do not know / prefer not to answer	

(Multiple responses possible)

Please specify which other work clothing you have access to:

____

To what extent do you feel that your work clothing protects you against cold?

- 0 (Not at all)
- 1
- 2
- 3
- 4
- 5

- 6
- 7
- 8
- 9
- 10 (To a very high extent)

Do you feel that you can control how much time you spend outdoors during a working day?

- Completely agree
- Partly agree
- Agree to a small extent
- Do not agree at all
- Do not know / prefer not to answer

How do you usually experience thermal comfort when working outdoors in winter:

	Very cold	Slightly cold	Neutral	Slightly warm	Very warm	Do not know / prefer not to answer
**Whole body**						
**Face**						
**Hands**						
**Feet**						

The following questions concern today, when you carried out the measurements.

How did you perceive the outdoor temperature TODAY (when you were wearing the measuring equipment)?

- Much warmer than usual for the season
- Slightly warmer than usual for the season
- Typical for the season
- Slightly colder than usual for the season
- Much colder than usual for the season

- Do not know / prefer not to answer

Did you experience that TODAY's working day differed in any way from a typical working day?

- No, everything was as usual
- Yes, I was more physically active than usual
- Yes, I was more sedentary than usual
- Yes, I was indoors more than usual
- Yes, I was outdoors more than usual
- Other
- Do not know / prefer not to answer

Please elaborate:

____

What proportion of your working day do you estimate that you spent outdoors TODAY?

- Not at all
- One tenth of the time
- One quarter of the time
- Half of the time
- Three quarters of the time
- Almost all of the time
- Do not know / prefer not to answer

Did you experience any specific work task TODAY that was particularly physically demanding or challenging?

- No
- Yes
- Do not know / prefer not to answer

Please describe what you did:

____

Health-related questions

Have you been diagnosed by a physician with any of the following conditions?

	Yes	No	Do not know / prefer not to answer
High blood pressure			
Angina pectoris / myocardial infarction			
Asthma			
Chronic obstructive pulmonary disease (COPD)			
Rheumatic disease			
Diabetes			
Osteoarthritis			

Have you ever suffered from a local cold injury (frostbite)?

	Yes	No	Do not know / prefer not to answer
Face			
Hands			
Feet			
Other body part			

Do you experience any of the following symptoms when exposed to cold?

Shortness of breath	
Cough	
Wheezing	
Increased mucus production in the airways	
Nasal congestion	
Runny nose	
Chest pain or pressure	
Irregular or rapid heartbeat	
Pain in neck/shoulders	
Pain in arms	
Pain in thoracic or lumbar spine	
Knee pain	
Hip pain	
Visual disturbances	

Headache	
Other	
None of the above	
Do not know / prefer not to answer	

(Multiple responses possible)

Please specify:

____

Cold sensitivity in the hands is defined as a collection of acquired symptoms (pain, altered sensation, stiffness or colour changes) that result in avoidance of cold. Do you experience such symptoms?

- Not at all
- Hardly at all
- To a small extent
- To a moderate extent
- To a great extent
- Do not know / prefer not to answer

The image below shows white fingers, also known as Raynaud's phenomenon.

Does one or several of you fingers turn white (as in the picture) when you are exposed to moisture or cold?

- Yes
- No
- Do not know / prefer not to answer

Does working in cold conditions negatively affect any of the following factors?

	**Not at all**	**Hardly at all**	**To a small extent**	**To a moderate extent**	**To a great extent**	**Do not know / prefer not to answer**
Concentration						
Endurance						
Mobility						
Strength						

Speed						

Is there any additional comment you would like to provide?

____
